# Prospective comparative assessment of liver stiffness and steatosis between iLivTouch and FibroScan

**DOI:** 10.1097/HC9.0000000000001066

**Published:** 2026-10-19

**Authors:** Frida Nuñez-Mercado, Eva Juárez-Hernández, Raúl A Gómez-Mendoza, Martha H. Ramos-Ostos, Misael Uribe, Graciela Castro-Narro, Iván López-Méndez

**Affiliations:** 1 Translational Research Unit, Medica Sur Clinic and Foundation, Mexico City, Mexico; 2 Gastroenterology and Obesity Unit, Medica Sur Clinic and Foundation, Mexico City, Mexico; 3 Integral Diagnosis and Treatment Unit, Medica Sur Clinic and Foundation, Mexico City, Mexico; 4 Hepatology and Transplants Unit, Medica Sur Clinic and Foundation, Mexico City, Mexico

**Keywords:** attenuation parameter, Bland–Altman, liver, obesity

## Abstract

**Background:**

Metabolic dysfunction–associated steatotic liver disease currently represents the leading cause of chronic liver disease worldwide. FibroScan and iLivTouch are 2 devices that employ similar principles to estimate liver steatosis and fibrosis, although few studies have directly compared their results.

**Aim:**

To evaluate the concordance of 2 transient elastography devices for the estimation of liver steatosis and fibrosis.

**Methods:**

A prospective study was conducted. Each participant underwent liver elastography with both devices consecutively under standardized conditions. Clinical, anthropometric, and biochemical variables were collected. The diagnostic accuracy of iLivTouch was calculated using FibroScan and noninvasive scores as references.

**Results:**

A total of 1004 patients were enrolled, with a median body mass index of 25.7 kg/m^2^ [IQR=23.6–28.7]. Median of attenuation values was 250 dB/m [IQR=214–291] with FibroScan, 262 dB/m [IQR=239–310] with iLivTouch. Median liver stiffness measurements were 4.1 kPa [IQR=3.4–4.8] for FibroScan and 6.2 kPa [IQR=5.5–7.1] for iLivTouch. Bland–Altman analysis revealed mean differences of −19.45±49.62 dB/m for steatosis and −2.43±2.51 kPa for stiffness. Using FibroScan (>269 dB/m) as the reference, iLivTouch ≥244 dB/m demonstrated 90% sensitivity for detecting liver steatosis. The correlation between the FAST scores of the 2 devices was very strong (rho=0.90, *p*≤0.0001).

**Conclusion:**

iLivTouch serves as an effective alternative to assess liver fibrosis and steatosis in the general population, including overweight and obese individuals.

## Graphical abstract

**Figure:**
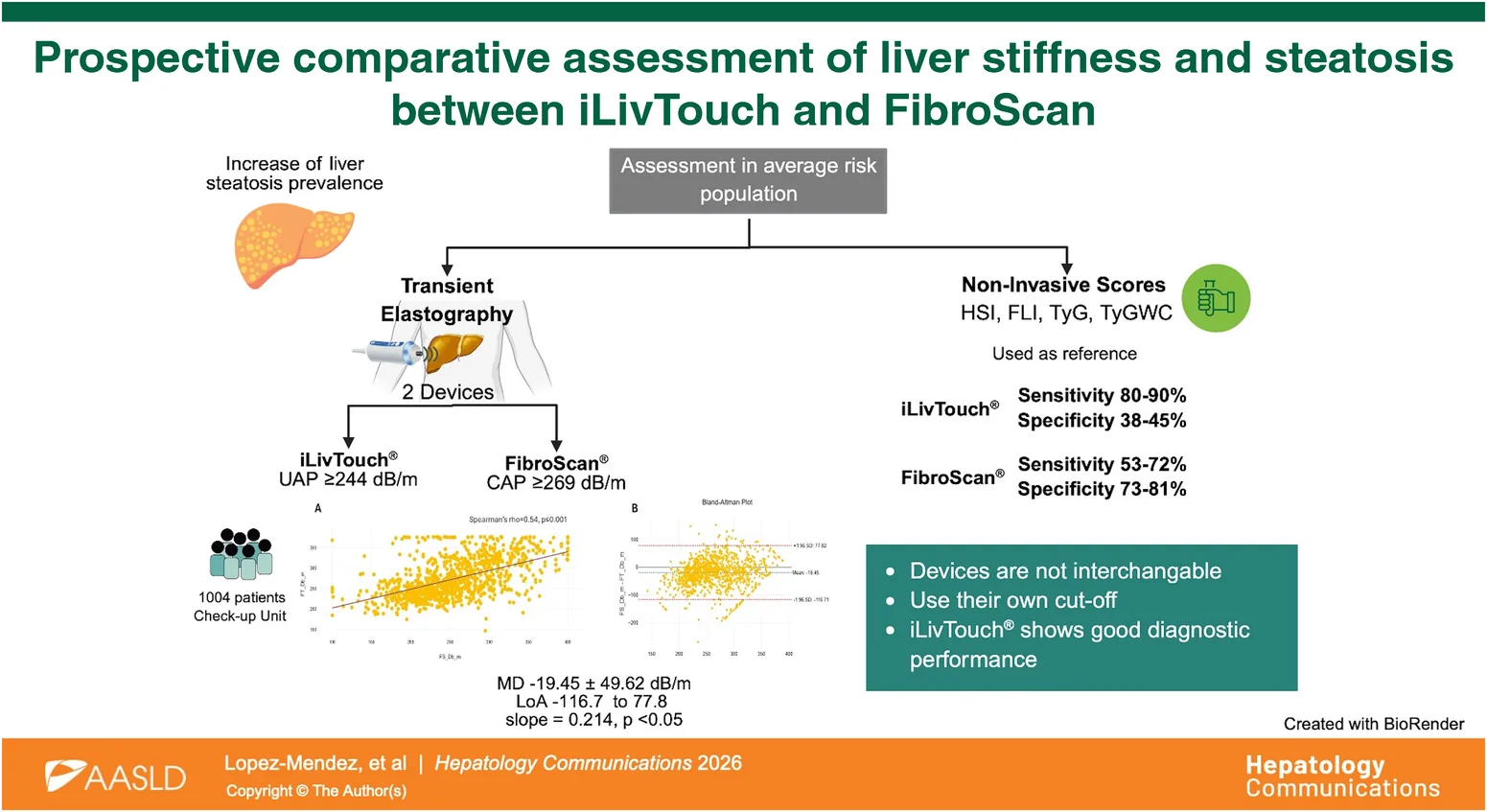

## INTRODUCTION

Metabolic dysfunction–associated steatotic liver disease (MASLD) is a metabolic disorder characterized by hepatic fat accumulation (>5%) in the presence of at least one metabolic abnormality; it excludes those patients with excessive alcohol consumption, those with hepatotoxic drug treatment, and those with liver steatosis secondary to other known etiologies.^1^

MASLD is strongly associated with metabolic syndrome, as insulin resistance plays a pivotal role in the pathophysiology of both conditions.^2^,^3^ MASLD can progress to liver fibrosis, becoming a leading cause of liver cirrhosis and hepatocellular carcinoma. Currently, MASLD is the most prevalent form of chronic liver disease worldwide.^4^ Its global prevalence ranges from 17% to 46%, with higher rates reported in the Middle East (31%) and Latin America (30%),^5^ largely due to the high incidence of predisposing factors such as type 2 diabetes mellitus, obesity, metabolic syndrome, and high prevalence of *PNPLA3* polymorphism, particularly in Hispanic populations.^6^

Diagnosis of MASLD and estimation of liver fibrosis require confirmation of steatosis and fibrosis, with liver biopsy serving as the reference standard. However, owing to its invasive nature, various noninvasive methods, such as imaging techniques and noninvasive scores, have been developed and validated. The use of noninvasive tools is recommended in primary care/non-hepatology and hepatology settings due to their relative accessibility, low cost (for scores that use biochemical indicators), and, unlike biopsy, their ability to be repeated.^7^ Among the noninvasive scores, the Fatty Liver Index (FLI), Hepatic Steatosis Index (HSI), and Triglyceride-Glucose Index (TyG), as well as their combinations, have demonstrated good diagnostic accuracy for hepatic steatosis, with reported sensitivities ranging from 45% to 95%.^8^ For imaging, ultrasound is the most commonly used screening tool, with a sensitivity of 80%–90%. Nevertheless, its operator-dependent variability has prompted the evaluation of more advanced modalities such as magnetic resonance imaging (MRI) and computed tomography (CT), which exhibit higher diagnostic accuracy, with sensitivities of 88% and 95%, respectively. However, access to these advanced techniques remains limited.^9^ For fibrosis assessment, the fibrosis-4 index (FIB-4) and aspartate aminotransferase-to-platelet ratio index (APRI) scores demonstrate the highest diagnostic accuracy for ruling out significant fibrosis, with negative predictive values (NPVs) of 90% and 76.2%, respectively.^10^ MRI also shows high sensitivity (90%) for diagnosing fibrosis in MASLD patients, although its availability is restricted.^11^

Transient elastography (TE) is a noninvasive imaging method capable of simultaneously estimating both liver steatosis and fibrosis with good diagnostic accuracy.^12^ TE employs low-frequency ultrasound waves to measure liver stiffness (in kPa) based on shear wave velocity and to estimate hepatic fat content through ultrasound signal attenuation (in dB/m).^13^ It has a failure or non-interpretation rate of 5%–15%. Key advantages of TE include its noninvasiveness, rapid execution, ease of use, and utility in both screening and diagnostic settings.^14^ Despite limitations such as reduced diagnostic performance in the presence of hepatic inflammation, obesity, or ascites,^14^ TE has been incorporated into clinical guidelines from Europe, the Americas, Latin America, and the Asia-Pacific region as a validated method for assessing steatosis and fibrosis.

The first TE device, FibroScan, was developed in France by Echosens, Paris, France,^15^ and became commercially available in 2003. In 2013, Wuxi Hisky Medical Technology Co., Ltd. (Wuxi, China) introduced its TE device, iLivTouch, which operates on the same physical principles as those used to quantify liver steatosis and fibrosis. The diagnostic accuracy of iLivTouch has been evaluated against histological reference standards, demonstrating a success rate of 96.5% and area under the receiver operating characteristic curve (AUROC) values of 0.88–0.93 for steatosis and 0.71–0.77 for fibrosis.^16^

The present study aimed to evaluate the concordance of liver steatosis and fibrosis measurements between 2 TE devices, namely FibroScan and iLivTouch, in a population at average risk for hepatic steatosis.

## METHODS

### Study population

This prospective study consecutively enrolled patients aged 18 years or older who attended routine health check-ups at the Integral Diagnostic and Treatment Unit of Medica Sur Clinic & Foundation. Clinical data, anthropometric measurements, and fasting biochemical parameters were collected. Exclusion criteria included a history or current diagnosis (confirmed by laboratory testing and medical history during the visit) of chronic liver diseases (autoimmune, viral, or drug-induced), significant abnormalities in liver function test (more than 2.5-fold the upper limit of the normal range), human immunodeficiency virus infection, hepatotoxic medication use, active malignancy, pregnancy, and significant alcohol consumption (>20 g/day in women and >30 g/day in men). The study was conducted in accordance with the Declaration of Helsinki, and all participants provided written informed consent. The study protocol was approved by the Institutional Ethics Committee of MEDICA SUR S.A.B. DE C.V. (approval number 2024-EXT-828; March 15, 2024).

### Liver steatosis and fibrosis assessment

TE was performed sequentially, without specific or randomized order, using FibroScan (FibroScan 502 Touch, Echosens, Paris, France) and iLivTouch (iLivTouch-FT100, Wuxi Hisky Med, China) in all participants by a single certified operator per device by following the manufacturer’s recommendations, including a minimum 4-hour fasting period. Measurements were obtained from the right liver lobe through the intercostal spaces with the patient in the supine position. Patients whose TE studies failed to meet reliability criteria, defined as at least 10 valid measurements, an interquartile range (IQR) of stiffness <30% of the median, and an IQR of attenuation <40 dB/m, were excluded. Liver steatosis was defined using device-specific attenuation cutoff values: ≥269 dB/m for FibroScan^17^ and ≥244 dB/m for iLivTouch.^16^ Significant liver fibrosis was defined as a stiffness value ≥8.1 kPa on both devices.

In addition, noninvasive scores for liver steatosis, fibrotic metabolic dysfunction–associated steatohepatitis (MASH), and fibrosis were calculated for all patients using the following established formulas:

1. FLI=(e0.953∗loge(triglycerides)+0.139∗BMI+0.718∗loge(GGT)+0.053∗WC−15.745)/(1+e0.953∗loge (triglycerides)+0.139∗BMI+0.718∗loge(GGT)+0.053∗WC−15.745) ×100.^18^ Cutoff value: ≥60.
2. HSI=8 × ALT/AST + BMI (+2 if type 2 DM present, +2 if female).^19^ Cutoff value: ≥36.
3. TyG=Ln [Tg (mg/dL) × fasting glucose (mg/dL)/2].^20^ Cutoff value: ≥4.61.^21^
4. TyG-WC=(TyG index) × (waist circumference (cm)).^22^ Cutoff value: ≥425.6.^21^
5. FIB-4=(Age × AST)/(Platelets × √(ALT)).^23^ Cutoff value: ≥1.45.
6. APRI=(AST in IU/L)/(AST upper limit of normal in IU/L)/(Platelets in 10^9^/L).^24^ Cutoff value: ≥0.7.
7. FAST=e(−1.65+1.07×ln(LSM)+2.66∗10^−8^∗CAP^3-63.3^×AST^−1^)/1+e (−1.65+1.07×ln(LSM)+2.66∗10^−8^∗CAP^3-63.3^×AST^−1^).^25^ Cutoff value: <0.35 rules out significant fibrosis or inflammation, 0.35–0.67 is inconclusive, and >0.67 indicates significant fibrosis and inflammation.

### Statistical analysis

Normality was assessed using the Kolmogorov–Smirnov test; data were non-normally distributed. Thus, continuous variables are reported as medians with interquartile ranges (IQRs), and categorical variables as frequencies and percentages.

Correlation between dB/m and kPa measurements from the 2 devices was evaluated using the Spearman rank correlation coefficient. Bland–Altman analysis was performed to assess agreement between FibroScan and iLivTouch for both attenuation and stiffness, with limits of agreement (LoA) defined as the mean difference±1.96 times the standard deviation of the differences. Proportional bias was also examined. A univariate analysis was performed, and those variables with a *p*-value <0.01 were included in a multivariate logistic regression to identify factors associated with discrepancies between the 2 devices, with discrepant studies defined as those outside the LoA.

The diagnostic accuracy of iLivTouch and FibroScan for steatosis was evaluated using noninvasive scores as reference standards. Sensitivity, specificity, positive and negative predictive values, area under the curves (AUCs), and likelihood ratios were calculated. In addition, FAST scores derived from each device were correlated. Analyses were conducted for the entire cohort and subsequently restricted to patients with overweight or obesity (BMI ≥25 kg/m^2^). Statistical analyses were performed using IBM SPSS Statistics for PC, version 20 (IBM Corp., Armonk, NY, USA), and the Numiqo Statistics Calculator (Numiqo Team (2025); numiqo: Online Statistics Calculator; numiqo e.U. Graz, Austria. URL https://numiqo.es).

## RESULTS

### Patients’ characteristics

From March 2024 to August 2025, 1112 patients were screened, and 1004 patients were enrolled in the study (Supplemental Figure S1, https://links.lww.com/HC9/C505); 59.5% (n=597) were males, with a median age of 47 years [IQR 40–54] and a median BMI of 25.7 kg/m^2^ [IQR 23.6–28.7]. Overweight or obesity (BMI ≥25 kg/m^2^) was present in 59% of participants (n=592). The prevalence of diabetes mellitus was 6.6% (n=66), and dyslipidemia was observed in 55.8% of participants (n=559). Baseline characteristics are summarized in Table 1, and characteristics of patients with BMI >25 kg/m^2^ are presented in Supplemental Table S1, https://links.lww.com/HC9/C505.

**TABLE 1:** General characteristics of all patients (n=1004)

All patients (n=1004)	
Variable	% (n)/M [IQR]
Male	59.5% (597)
Age (y)	47 [40–54]
BMI (kg/m^2^)	25.7 [23.6–28.7]
WC (cm)	93 [87–99]
Fasting glucose (mg/dL)	90 [84–95]
Triglycerides (mg/dL)	111.5 [80.2–156]
GGT (UI/L)	23 [16–35]
AST (UI/L)	24 [20–30]
ALT (UI/L)	26 [19–36]
Platelets (×10^3^/µL)	269 [233–312]
HbA1c (%)	5.5 [5.4–5.8]
Liver steatosis and fibrosis assessment	
dB/m, FibroScan	250 [214.8–291]
dB/m, iLivTouch	262 [239–310]
IQR, dB/m, FibroScan	26 [20–32]
IQR, dB/m, iLivTouch	15 [10–23]
LS, FibroScan (>269 dB/m)	37.8% (380)
LS, iLivTouch (>244 dB/m)	68.6% (689)
kPa, FibroScan	4.1 [3.4–4.8]
kPa, iLivTouch	6.2 [5.5–7.1]
IQR, kPa, FibroScan	12 [9–16]
IQR, kPa, iLivTouch	8 [4–14]
Fibrosis, FibroScan (>8.1 kPa)	0.3% (3)
Fibrosis, iLivTouch (>8.1 kPa)	14.3% (144)
LS by HSI	44.8% (450)
LS by FLI	23.9% (240)
LS by TyG	50.9% (511)
LS by TyG-WC	51.3% (515)
LF by FIB-4	9.9% (99)
LF by APRI	2.1% (21)

Abbreviations: ALT, alanine aminotransferase; APRI, aspartate aminotransferase-to-platelet ratio index; AST, aspartate aminotransferase; BMI, body mass index; FIB-4, fibrosis-4 index; FLI, fatty liver index; GGT, gamma-glutamyl transferase; HbA1c, glycated hemoglobin; HSI, hepatic steatosis index; IQR, interquartile range; LF, liver fibrosis; LS, liver steatosis; TyG, triglyceride-glucose index; TyG-WC, triglyceride-glucose index–waist circumference; WC, waist circumference.

### Liver steatosis and fibrosis determination

The median attenuation was 250 dB/m [IQR 214–291] with FibroScan and 262 dB/m [IQR 239–310] with iLivTouch. Using device-specific cutoff values, steatosis prevalence was 37.8% (n=380) with FibroScan and 68.6% (n=689) with iLivTouch. Noninvasive scores yielded steatosis prevalence ranging from 23% to 51%, with the highest rate observed using the TyG-WC index (51.3%, n=515) (Table 1).

Median stiffness values were 4.1 kPa [IQR 3.4–4.8] for FibroScan and 6.2 kPa [IQR 5.5–7.1] for iLivTouch, corresponding to significant fibrosis (≥8.1 kPa) prevalence of 0.3% (n=3) and 14.3% (n=144), respectively (Table 1). Advanced fibrosis (≥12 kPa) was detected in 0.3% (n=3) with FibroScan and 3.2% (n=33) with iLivTouch. Noninvasive scores indicated significant fibrosis in 9.9% (n=99) by FIB-4 and 2.1% (n=21) by APRI. Regarding the FAST score, 95% (n=955) of patients were classified as having no significant fibrosis or inflammation using FibroScan data, compared with 86.9% (n=872) using iLivTouch data.

In overweight and obese patients (n=592), median attenuation values were 273 dB/m [IQR 235–310] for FibroScan and 281 dB/m [IQR 249–326] for iLivTouch, with steatosis prevalence of 53.4% (n=316) and 80.6% (n=477), respectively. Median stiffness and fibrosis prevalence were also higher with iLivTouch (6.5 kPa [IQR 5.7–7.5], 20.3% [n=120]) than with FibroScan (4.2 kPa [IQR 3.6–5.0], 0.5% [n=3]). Advanced fibrosis was observed in 0.5% (n=3) with FibroScan and 5.5% (n=31) with iLivTouch. Among noninvasive scores, the TyG-WC index showed the highest steatosis prevalence (70.3%, n=416), while FIB-4 indicated greater fibrosis prevalence (9.1%, n=54) than APRI (2.4%, n=14). According to the FAST score, 92.2% (n=546) and 80.7% (n=478) of overweight/obese patients were classified as having no significant fibrosis or inflammation using FibroScan and iLivTouch data, respectively.

### Concordance between FibroScan and iLivTouch in liver steatosis estimation

Bland–Altman analysis for the full cohort revealed a mean difference of −19.45±49.62 dB/m, with LoA of −116.7 [95% CI −122 to −111.4] to 77.8 [95% CI 72.5 to 83.1], and significant proportional bias (slope=0.214, *p*<0.05) (Figure 1). In the correlation analysis, we observed rho=0.54 (*p*≤0.0001).

**FIGURE 1:**
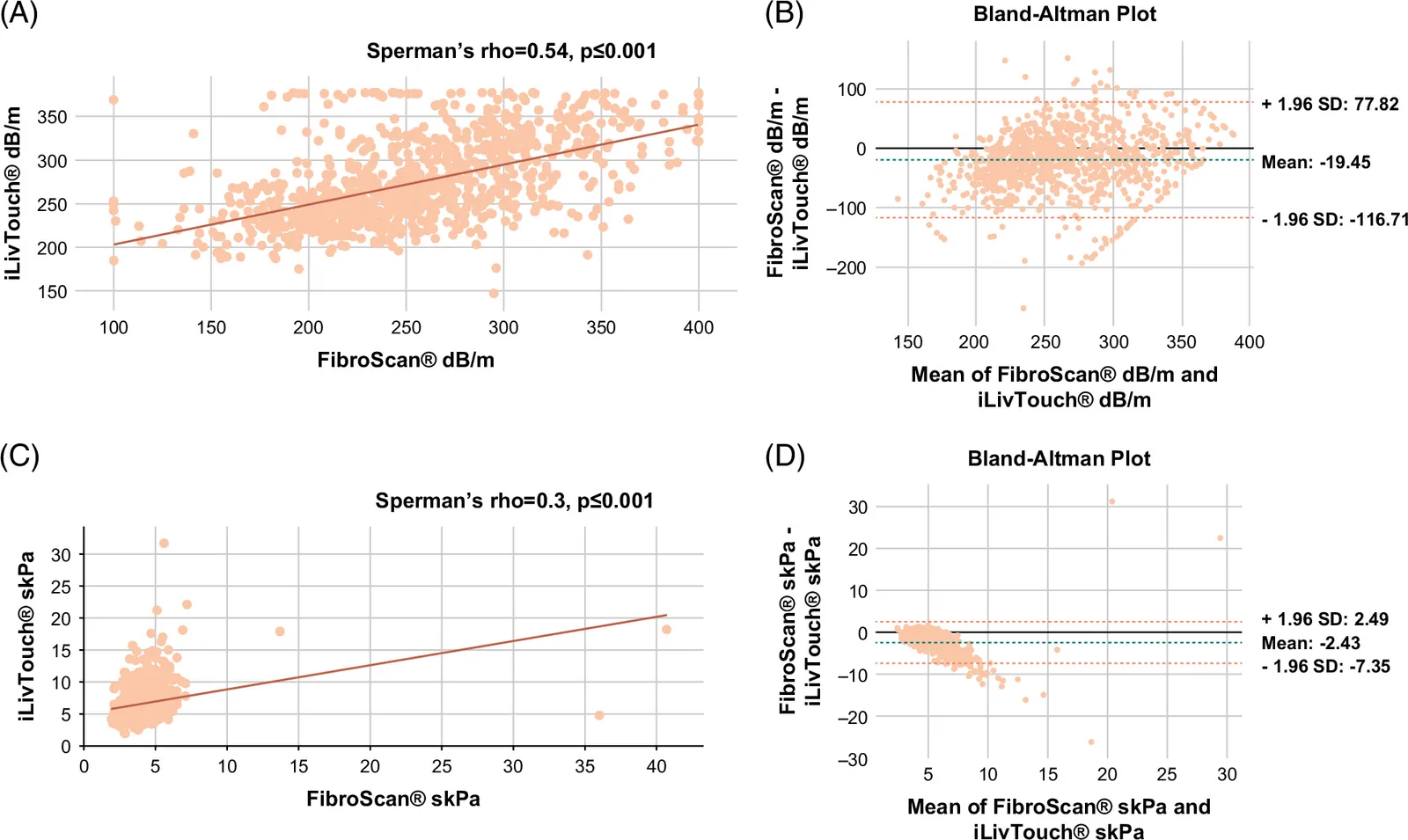
Correlation and Bland–Altman analyses assessing concordance between FibroScan and iLivTouch in estimating liver steatosis and fibrosis across all patients.

The proportional bias result indicates that the discrepancy between the devices decreases at higher values. Therefore, the LoA were calculated using regression adjustment, resulting in −32.7 [95% CI −128.1 to +62.7] for the 5th percentile (median 202* *dB/m), −20.7 [95% CI −116.1 to +74.7] for the 50th percentile (median 258* *dB/m), and −1.8 [95% CI −97.2 to +93.6] for the 95th percentile (median 345.9* *dB/m).

In overweight and obese patients, Bland–Altman analysis showed a mean difference of 13.56±53.28* *dB/m, LoA of −117.86 [95% CI −125.4 to −110.5] to 90.87 [95% CI 83.4–98.3] (Figure 2), and proportional bias (slope=0.164, *p*=0.0012). The adjusted LoA showed a bias of −24.2 [95% CI −127.7 to +79.3] in the 5th percentile (median 217 dB/m); −13.7 [95% CI −117.2 to +89.7] for the 50th percentile (median 280 dB/m), and for the 95th percentile (median 357 dB/m), the bias was −1.2 [95% CI −104 to +102.3].

**FIGURE 2:**
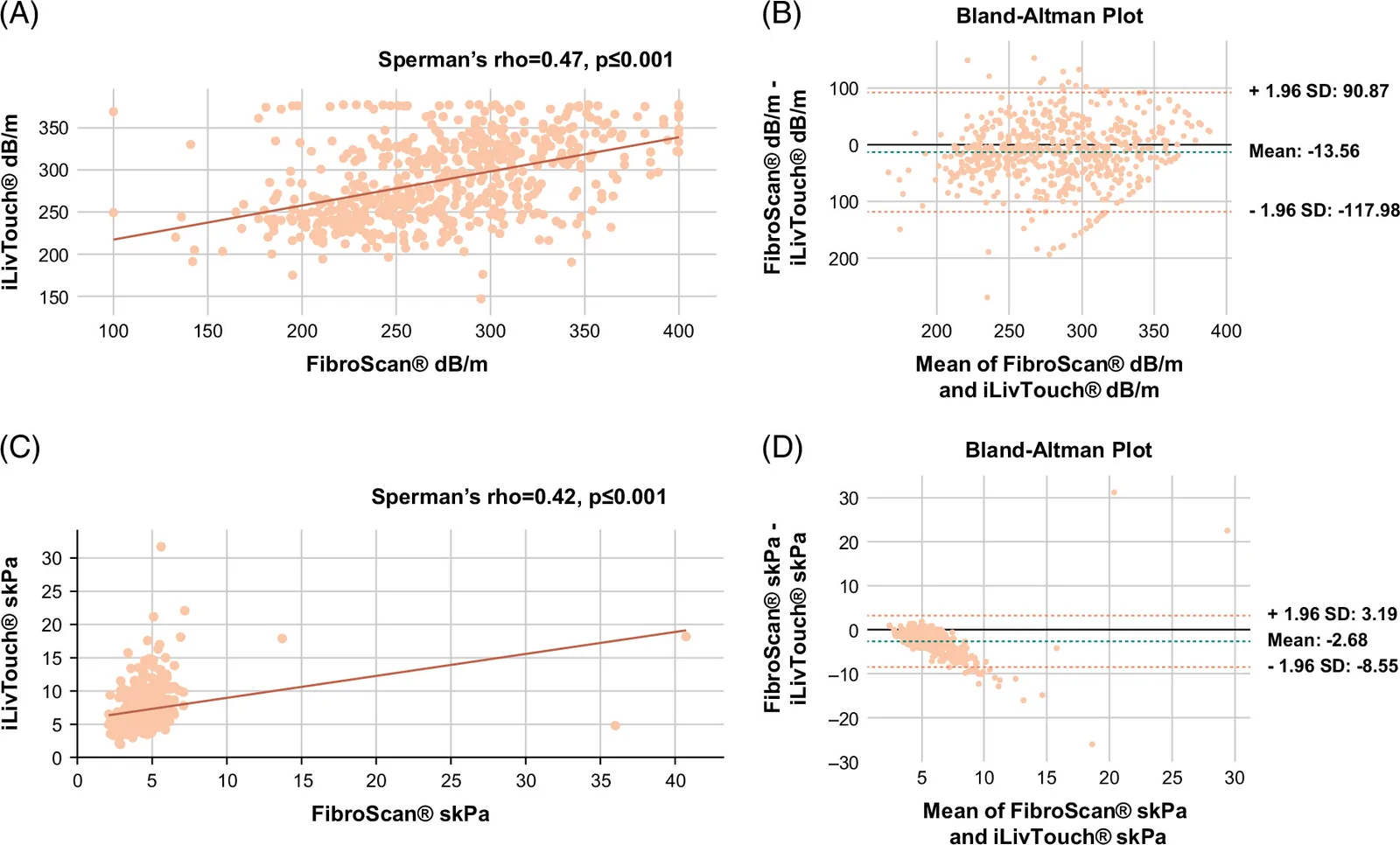
Correlation and Bland–Altman analyses in overweight and obese patients assessing concordance between FibroScan and iLivTouch for liver steatosis and fibrosis estimation.

### Concordance between FibroScan and iLivTouch in liver fibrosis estimation

For fibrosis estimation across all patients, the Spearman correlation was rho=0.3 (*p*<0.001). Bland–Altman analysis showed a mean difference of −2.43±2.51 kPa, LoA of −7.35 [−7.62 to −7.09] to 2.49 [2.2–2.76], and proportional bias (slope=−0.37, *p*≤0.001) (Figure 1). The discrepancy increases with higher liver stiffness, with adjusted LoA of −1.79 [−6.56 to +2.98] for the 5th percentile (median 3.7 kPa); −2.32 [−7.09 to +2.45] for the 50th percentile (median 5.2 kPa); and −3.35 [−8.12 to +1.42] for the 95th percentile (median 7.9 kPa). Prevalence of discrepant studies was 2.3% (n=23).

In overweight and obese patients, the correlation between kPa values was rho=0.42 (*p*<0.001). Bland–Altman analysis yielded a mean difference of −2.68±2.99 kPa, LoA of −8.55 [95% CI −8.96 to −8.12] to 3.19 [95% CI 2.76 to 3.60], and proportional bias (slope=−0.28, *p*<0.001) (Figure 2). The adjusted LoA was −2.16 [95% CI −7.93 to + 3.60] for the 5th percentile (median 3.9 kPa); −2.58 [95% CI −8.35 to +3.18] for the 50th percentile (median 5.4 kPa), and for the 95th percentile (median 8.5 kPa) the bias was −3.44 [95% CI −9.20 to +2.33]. Prevalence of discrepant studies was 2.7% (n=16).

### Factors associated with the discrepancy between iLivTouch and FibroScan

Discrepant studies were defined as those outside the regression-adjusted LoA; according to this classification, the prevalence of discrepancy in steatosis assessment in all patients was 5.7% (n=56). In univariate analysis, significant association was observed in age (*p*=0.03), AST (*p*=0.02), IQR dB/m of iLivTouch, and IQR kPa of iLivTouch; in multivariate analysis independent factors associated to discrepancy were IQR dB/m of iLivTouch (OR 0.93, 95% CI 0.90–0.97, *p*<0.001), and IQR kPa of iLivTouch (OR 1.05, 95% CI 1.02–1.09, *p*<0.001) (Table 2).

**TABLE 2:** Factors associated with dB/m discrepancy between iLivTouch and FibroScan in all patients (n=1004)

	Univariate		Multivariate	
Variable	OR (95% CI)	*p*	OR (95% CI)	*p*
Male	1.05 (0.60–1.83)	0.84		
Age (y)	1.02 (1.00–1.05)	0.03		
BMI (kg/m^2^)	1.02 (0.96–1.83)	0.49		
WC (cm)	0.99 (0.96–1.01)	0.52		
DM	1.09 (0.38–3.12)	0.86		
HTN	1.62 (0.83–3.15)	0.15		
Dyslipidemia	1.45 (0.82–2.54)	0.19		
Smoking	1.2 (0.57–2.50)	0.62		
FG (mg/dL)	1.00 (0.97–1.02)	0.98		
Triglycerides (mg/dL)	1.01 (0.99–1.00)	0.44		
GGT (UI/L)	1.00 (0.99–1.01)	0.26		
AST (UI/L)	1.02 (1.00–1.04)	0.02		
ALT (UI/L)	1.01 (1.00–1.03)	0.05		
Platelets (×10^3^/µL)	0.99 (0.99–1.00)	0.40		
HbA1c (%)	1.20 (0.80–1.81)	0.37		
IQR, dB/m, FibroScan	1.01 (0.98–1.05)	0.29		
IQR, kPa, FibroScan	1.00 (0.95–1.05)	0.95		
IQR, dB/m, iLivTouch	0.92 (0.89–0.96)	<0.0001	0.93 (0.90–0.97)	<0.001
IQR, kPa, iLivTouch	1.07 (1.03–1.10)	<0.0001	1.05 (1.02–1.09)	<0.001

Abbreviations: ALT, alanine aminotransferase; AST, aspartate aminotransferase; BMI, body mass index; CI, confidence interval; DM, diabetes mellitus; FG, fasting glucose; GGT, gamma-glutamyl transferase; HbA1c, glycated hemoglobin; HTN, hypertension; IQR, interquartile range; OR, odds ratio; WC, waist circumference.

The independent association of IQR dB/m of iLivTouch can be explained by the fact that, given the high variability of the dB signal, the device tends to average toward the center of the range, thus reducing the likelihood of falling outside the adjusted LoA. On the other hand, regarding the IQR kPa of iLivTouch, greater measurement variability increases the risk of discrepancy between devices.

In overweight and obese patients, the prevalence of discrepancy was 5.5% (n=33). In univariate analysis, significant association were observed in IQR dB/m of iLivTouch (OR 0.94, 95% CI 0.90–0.99, *p*=0.01) and in IQR kPa of iLivTouch (OR 1.04, 95% CI 1.00–1.09, *p*=0.02), and once again this variables presented significant association in same direction than in all patients in multivariate analysis: IQR dB/m of iLivTouch (OR 0.95, 95% CI 0.90–0.99, *p*=0.03), and IQR kPa of iLivTouch (OR 1.04, 95% CI 1.0–1.08, *p*=0.04), however, it is important to highlight that only observed 33 cases.

### Diagnostic accuracy of iLivTouch and FibroScan with noninvasive scores for liver steatosis as a reference

The diagnostic accuracy of both devices, using noninvasive scores as a reference, was evaluated, with FibroScan generally showing greater specificity (73%–81%) and AUC, while iLivTouch exhibits higher sensitivity (80%–90%). The best diagnostic performance is observed when using the FLI as a reference, with a sensitivity of 90.0% (95% CI 85.6–93.2) and specificity of 38.1% (95% CI 34.7–41.6) for iLivTouch and a sensitivity of 72.9% (95% CI 67.0–78.1) and specificity of 73.2% (95% CI 69.9–76.2) for FibroScan (Table 3).

**TABLE 3:** Diagnostic accuracy of iLivTouch and FibroScan in all patients and in overweight and obese patients, using noninvasive scores as a reference standard

		All patients (n=1004)						
Reference method	TE device^a^	Sen %, 95% CI	Spe %, 95% CI	PPV, %	NPV, %	+LR	−LR	AUC
HSI ≥36	iLivTouch	83.8 (80.1–86.9)	43.7 (39.6–47.8)	54.7	76.8	1.49	0.37	0.73
	FibroScan	61.1 (56.5–65.5)	81.0 (77.6–84.1)	72.4	72	3.22	0.48	0.78
TyG	iLivTouch	82.0 (78.4–85.1)	45.2 (40.9–49.6)	60.8	70.8	1.50	0.40	0.69
	FibroScan	53.2 (48.9–57.5)	78.1 (74.2–81.5)	71.6	61.7	2.43	0.60	0.73
TyG-WC	iLivTouch	80.0 (76.3–83.2)	43.4 (39.0–47.8)	59.8	67.3	1.41	0.46	0.68
	FibroScan	53.4 (49.1–57.7)	78.5 (74.7–81.9)	72.4	61.5	2.49	0.59	0.73
FLI ≥60	iLivTouch	90.0 (85.6–93.2)	38.1 (34.7–41.6)	31.3	92.4	1.45	0.26	0.76
	FibroScan	72.9 (67.0–78.1)	73.2 (69.9–76.2)	46.1	89.6	2.72	0.37	0.81
		**Overweight and obese patients (n=592)**						
		**Sen %, 95% CI**	**Spe %, 95% CI**	**PPV, %**	**NPV, %**	**+LR**	**−LR**	**AUC**
HSI ≥36	iLivTouch	85.2 (81.5–88.3)	30.8 (24.4–38.1)	75.1	46.1	1.23	0.48	0.66
	FibroScan	63.6 (58.9–68.0)	71.5 (64.4–77.7)	84.5	44.6	2.23	0.51	0.72
TyG	iLivTouch	87.5 (83.8–90.4)	32.1 (26.1–38.7)	70.2	58.3	1.29	0.39	0.64
	FibroScan	61.6 (56.7–66.4)	61.7 (55.0–68.0)	74.7	46.7	1.61	0.62	0.66
TyG-WC	iLivTouch	85.6 (81.9–88.6)	31.2 (24.9–38.4)	74.6	47.8	1.24	0.46	0.64
	FibroScan	59.9 (55.1–64.5)	61.9 (54.6–68.8)	78.8	39.5	1.57	0.65	0.65
FLI ≥60	iLivTouch	91.5 (87.2–94.4)	26.5 (22.2–31.3)	44.9	82.6	1.24	0.32	0.70
	FibroScan	74.4 (68.4–79.5)	60.3 (55.2–65.3)	55.1	78.3	1.87	0.42	0.74

^a^ Cutoff points: iLivTouch 244 dB/m; FibroScan 269 dB/m.

Abbreviations: AUC, area under the curve; CI, confidence interval; FLI, fatty liver index; HSI, hepatic steatosis index; LR, likelihood ratio; NPV, negative predictive value; PPV, positive predictive value; Sen, sensitivity; Spe, specificity; TE, transient elastography; TyG, triglyceride-glucose index; TyG-WC, triglyceride-glucose index–waist circumference.

When diagnostic accuracy analysis was performed in patients with BMI >25 kg/m^2^, the sensitivity of iLivTouch remained in a range of 85.6%–91.5%, and the specificity of both devices was lower than in the entire cohort, with ranges of 60.3%–71.5% for FibroScan and 26.5%–32.1% for iLivTouch (Table 3).

Due to the low prevalence of liver fibrosis in our cohort (0.3%), diagnostic accuracy analysis was not performed.

### Correlation of FAST score with FibroScan and iLivTouch data

FAST scores calculated using data from the 2 devices showed a very strong correlation in the full cohort (rho=0.901, *p*≤0.0001). In overweight and obese patients, a strong correlation (rho=0.88, *p*≤0.0001) was observed between FAST scores calculated from FibroScan and iLivTouch data.

## DISCUSSION

TE has been established as a high-yield method for detecting liver steatosis and fibrosis, primarily because of its practicality and robust diagnostic performance. Existing evidence on diagnostic performance indicates that both FibroScan and iLivTouch demonstrate high diagnostic accuracy compared with liver biopsy and other imaging modalities.

In a prospective study of patients with diabetes mellitus who underwent liver biopsy, Yu et al^26^ reported high diagnostic accuracy of iLivTouch for mild (AUROC 0.84), moderate (AUROC 0.88), and severe (AUROC 0.89) steatosis. For fibrosis estimation, iLivTouch yielded AUROCs of 0.76 for significant fibrosis (≥F2), 0.76 for F3, and 0.92 for F4. Mu et al^27^ compared iLivTouch with MRI in 110 MASLD patients and found AUROCs of 0.95 for mild steatosis (≥5%), 0.86 for moderate steatosis (≥10%), and 0.90 for severe steatosis (≥20% fat deposition).

While this evidence supports the accuracy of iLivTouch against reference standards, it is equally important to evaluate the agreement between the 2 commercially available TE devices for estimating liver steatosis and fibrosis.

Our results show a fair correlation (rho=0.54, *p*≤0.0001) between FibroScan and iLivTouch for liver steatosis estimation. Despite a mean difference of 19.45 dB/m (*p*≤0.0001), both devices similarly estimate the presence and severity of liver steatosis, and iLivTouch maintains good diagnostic accuracy compared with other noninvasive methods. However, the devices are not interchangeable, likely due to differences in the acquisition and processing signal algorithms of each device. On the other hand, despite differences in kPa and dB/m measurements, FAST scores derived from both devices showed a strong correlation.

Although both devices use the same underlying technology, their design differs: one requires 2 probes, while the other adapts to subcutaneous adipose tissue thickness. This difference may contribute to overestimation by iLivTouch, possibly due to variations in measurement depth and probe positioning. iLivTouch incorporates a two-dimensional image-guided system that automatically calibrates dynamic probes based on the thickness of the subcutaneous adipose tissue.^28^

It is important to highlight the difference in the prevalence of steatosis with each of the devices, which results in a systematic overestimation that can have implications for the diagnosis of the evaluated populations. Once again, it emphasizes that the devices are not interchangeable and that the results of studies conducted with each device should be analyzed and interpreted with caution, mainly due to the lack of universal cutoff points.

It is crucial to emphasize the distinction between the cutoff points used to estimate steatosis. It must be noted that, to date, there are no universally established cutoff points in dB/m measurements for the diagnosis of hepatic steatosis. The thresholds for FibroScan, which are supported by the most robust evidence and were utilized in this study (≥269 dB/m), are derived from the meta-analysis by Cao et al^17^ conducted exclusively in patients with fatty liver. This represents a significant advantage, as other proposed cutoff points are based on studies involving patients with diverse liver pathologies. Conversely, the cutoff point for iLivTouch (≥244 dB/m) has been proposed by the manufacturer, based on observed diagnostic accuracy compared with biopsy, and determined using the Youden index.^16^

These factors raise concerns about the generalizability of internal cutoff thresholds and device sensitivity in screening populations, suggesting that the methods are not directly interchangeable. Considering the variability in both algorithms and device calibration, it is imperative to utilize the specific cutoff points established for each device when detecting liver fibrosis and steatosis. Although universal cutoff points are not available, those determined for individual devices have been validated across various scenarios. Transferring these cutoff points to different devices may lead to unreliable measurements.

It is important to note that although correlation and Bland–Altman analyses were conducted to estimate fibrosis, the results should be interpreted with caution, given the low prevalence of fibrosis (0.3%–3%). This caution is supported by the behavior of the adjusted LoA, which shows increased discrepancy between the 2 devices at the 95th percentile. The number of patients at this percentile should be considered in relation to the observed prevalence of fibrosis.

Even with this low prevalence of fibrosis in our cohort, the overestimation of fibrosis assessed by iLivTouch was also reported in the study by Serra et al,^29^ which evaluated the concordance between iLivTouch and FibroScan in 39 patients and in liver phantoms as a controlled testing method. Bland–Altman analysis revealed that iLivTouch consistently overestimated stiffness values by a mean value of 3.1 kPa compared with Fibroscan. In contrast to the assessment of steatosis, where we established that the devices are not interchangeable, the evaluation of fibrosis shows a similar trend in the agreement of LSM measurements across devices, despite the methodological differences of each study. However, unlike steatosis, the cutoff point for determining significant fibrosis is the same on both devices (8.1 kPa). This reinforces the need to establish universal cutoff points for TE evaluation.

Regarding diagnostic accuracy, iLivTouch demonstrated high sensitivity for detecting hepatic steatosis when FibroScan was used as the reference, exceeding that of noninvasive scores. However, based on the specificity values obtained in both analyses, with all patients and those with BMI ≥25 kg/m^2^, the iLivTouch diagnostic performance makes it a good noninvasive method for ruling out liver steatosis in average-risk populations.

According to the results observed in the comparison of the diagnostic performance of both devices, using noninvasive scores as a reference, the sensitivity of the iLivTouch is systematically higher than that of FibroScan, and the opposite is true in the case of specificity; these trends can be explained by the difference in cutoff points for the determination of steatosis for each device. The variability among the different noninvasive scores highlights that the observed diagnostic accuracy depends on the score used as a reference and not on the properties of each device. Considering these results, the iLivTouch demonstrates good diagnostic performance in a screening population.

Our study has certain limitations, the main one being the lack of a reference standard, which, in the case of detecting hepatic steatosis, is liver biopsy. However, since our focus is on screening, having biopsies is not feasible. Nevertheless, elastography has sufficient evidence to demonstrate that it is an alternative tool to biopsy.^30^,^31^ With this in mind, although it is not an ideal comparison, it could be considered similar from a clinical perspective. Another limitation of our study is that the data come from a single center, which may limit generalizability.

Most of the studies that have evaluated the concordance between both devices have been performed in patients with chronic liver disease diagnostics; therefore, a major strength of our study is its focus on a population at average risk for liver disease; most existing evidence has been obtained from patients with established MASLD or other chronic liver conditions. The results of our study have important implications for clinical practice. The correlation in steatosis (dB/m) suggests that both devices can be used with reasonable confidence for screening, ruling out, or quantifying steatosis; however, our results show that the measurements from both devices are correlated but systematically biased, so caution is warranted when comparing absolute values. Therefore, direct comparison of dB/m values between devices is discouraged when monitoring the same patient over time, emphasizing the consistent use of the same device throughout their treatment and monitoring. Multicenter regression analysis is required to determine adjustment factors for kPa and dB/m to enable the devices to be interchangeable in clinical practice.

Future studies with larger, multicenter cohorts are necessary to validate these findings. In addition, device-specific cutoff values should be established by accounting for differences in measurement depth and performance timing between the devices.

## CONCLUSION

Although iLivTouch and FibroScan demonstrated a fair correlation in assessing hepatic steatosis, the 2 devices are not directly interchangeable. Nevertheless, using their own cutoff point, iLivTouch proves to be an alternative for assessing both hepatic steatosis in the general population and in overweight and obese patients.

## Supplementary Material
