# Supplementary material for: Prospective comparative assessment of liver stiffness and steatosis between iLivTouch and FibroScan
Source: Hepatol Commun. 2026 Oct 19;10(11):e1066. doi: 10.1097/HC9.0000000000001066 (PMC13682804; doi:10.1097/HC9.0000000000001066)
Supplement: Supplementary file 1 [file hc9-10-e1066-s001.docx]

**Supplementary Figure 1. Diagram of selected studies.**

Figure legend: IQR Interquartile Range, LFT Liver function test

**Supplementary Table 1. Characteristics of patients with BMI >25 kg/m^2^**

|  | ≥25 kg/m^2^ (n=592) |
| --- | --- |
| Variable | **%(n)/ M [IQR]** |
| Male | 67.4% (399) |
| Age (years) | 48 [41-54] |
| BMI (kg/m^2^) | 28 [26.3-30.1] |
| WC (cm) | 95 [93-102] |
| Fasting glucose (mg/dL) | 92 [87-98.1] |
| Triglycerides (mg/dL) | 130 [93.8-173.2] |
| GGT (UI/L) | 27 [19-41] |
| AST (UI/L) | 25[21-31] |
| ALT (UI/L) | 29 [21-40] |
| Platelets (x10^3^/µL) | 271 [235-316.2] |
| HbA1c (%) | 5.6 [5.4-5.8] |
| Liver Steatosis and Fibrosis Assessment | |
| dB/m FibroScan® | 273 [235 -310] |
| dB/m iLivTouch® | 281 [249-326] |
| IQR dB/m FibroScan® | 26[19-32] |
| IQR dB/m iLivTouch® | 15 [9-22] |
| LS FibroScan® (>269 dB/m) | 53.4% (316) |
| LS iLivTouch® (>244 dB/m) | 80.6% (477) |
| kPa FibroScan® | 4.2[3.6-5.0] |
| kPa iLivTouch® | 6.5[5.7-7.5] |
| IQR kPa FibroScan® | 12[9-15.2] |
| IQR kPa iLivTouch® | 15[9-22] |
| Fibrosis FibroScan® (>8.1 kPa) | 0.5% (3) |
| Fibrosis iLivTouch® (>8.1 kPa) | 20.3% (120) |
| LS by HSI | 70.9% (420) |
| LS by FLI | 39.5% (234) |
| LS by TyG | 64.7%(383) |
| LS by TyGWC | 70.3% (416) |
| LF by FIB4 | 9.1% (54) |
| LF by APRI | 2.4% (14) |
| BMI, Body Mass Index; WC, Waist Circumference; GGT, Gamma-glutamyl Transferase; AST, Aspartate Aminotransferase; ALT, Alanine Aminotransferase; HbA1c, Glycated Hemoglobin; LS, Liver Steatosis; HSI, Hepatic Steatosis Index; FLI, Fatty Liver Index; TyG, Triglycerides Glucose Index; LF, Liver Fibrosis; IQR, Interquartile Range | |
